# On the rising potential of interdisciplinary rehabilitation in neurological disorders: A mini-review

**DOI:** 10.1097/MD.0000000000041947

**Published:** 2025-03-21

**Authors:** Abdulhameed Tomeh, Abdul Hanif Khan Yusof Khan, Zalina Abu Zaid, King-Hwa Ling, Liyana Najwa Inche Mat, Hamidon Basri, Ahmad Luqman Md Pauzi, Muhammad Hibatullah Romli, Wan Aliaa Wan Sulaiman

**Affiliations:** a Department of Neurology, Faculty of Medicine and Health Sciences, Universiti Putra Malaysia, Serdang, Selangor, Malaysia; b Laboratory of Medical Gerontology and Gerontechnology, Malaysian Research Institute on Ageing (MyAgeing^TM^), Universiti Putra Malaysia, Serdang, Selangor, Malaysia; c Department of Dietetics, Faculty of Medicine and Health Sciences, Universiti Putra Malaysia, Serdang, Selangor, Malaysia; d Department of Dietetics, Hospital Sultan Abdul Aziz Shah, Serdang, Selangor, Malaysia; e Department of Biomedical Sciences, Faculty of Medicine and Health Sciences, Universiti Putra Malaysia, Serdang, Selangor, Malaysia; f Department of Medicine, Faculty of Medicine and Health Sciences, Universiti Putra Malaysia, Serdang, Selangor, Malaysia; g Department of Rehabilitation Medicine, Faculty of Medicine and Health Sciences, Universiti Putra Malaysia, Serdang, Selangor, Malaysia.

**Keywords:** chronic pain, interdisciplinary rehabilitation, neurorehabilitation, stroke, traumatic brain injury

## Abstract

Collaboration among health and non-health professionals is growing exponentially as we approach the personalized medicine era, where the intervention plan is tailored according to the patient’s needs. This collaboration aims to develop highly efficient, patient-centered, holistic approaches, rather than singular interventions. Interdisciplinary rehabilitation is a rising theme to coordinate the efforts of various professionals, with the ultimate goal of increasing rehabilitants’ satisfaction and improving their overall quality of life. A typical rehabilitation team may comprise a rehabilitation physician, rehabilitation nurse, occupational therapist, physiotherapist, speech and language therapist, clinical psychologist, social worker, prosthetist, orthotist, rehabilitation engineer, and dietician. The need for inclusion of additional professions in the rehabilitation team is dynamic and varies depending on the population and health condition. Recently, various countries have begun incorporating interdisciplinary rehabilitation models into their healthcare frameworks. For example, the U.S. Veterans Affairs Polytrauma Rehabilitation Centers have set a precedent for integrating interdisciplinary approaches into neurological rehabilitation, while European nations such as Germany and Sweden have successfully implemented stroke and pain rehabilitation programs. Although interdisciplinary rehabilitation has demonstrated effectiveness in improving patient outcomes, further research is required to explore its long-term benefits, cost-effectiveness, and adaptability in resource-limited settings. In this mini-review, we summarize the current evidence on employing interdisciplinary rehabilitation in patients with neurological disorders and highlight the implications for future research and clinical practice.

## 1. Introduction

The World Health Organization defines rehabilitation as “a set of measures that assist individuals who experience, or are likely to experience, disability to achieve and maintain optimal functioning in interaction with their environments.”^[1]^ The process of rehabilitation has long been an integral part of the management of medical conditions, especially in patients with neurological disabilities, that is, neurorehabilitation, due to the limited regenerative capacity of the central nervous system after neurological insults.

**Figure 1.**
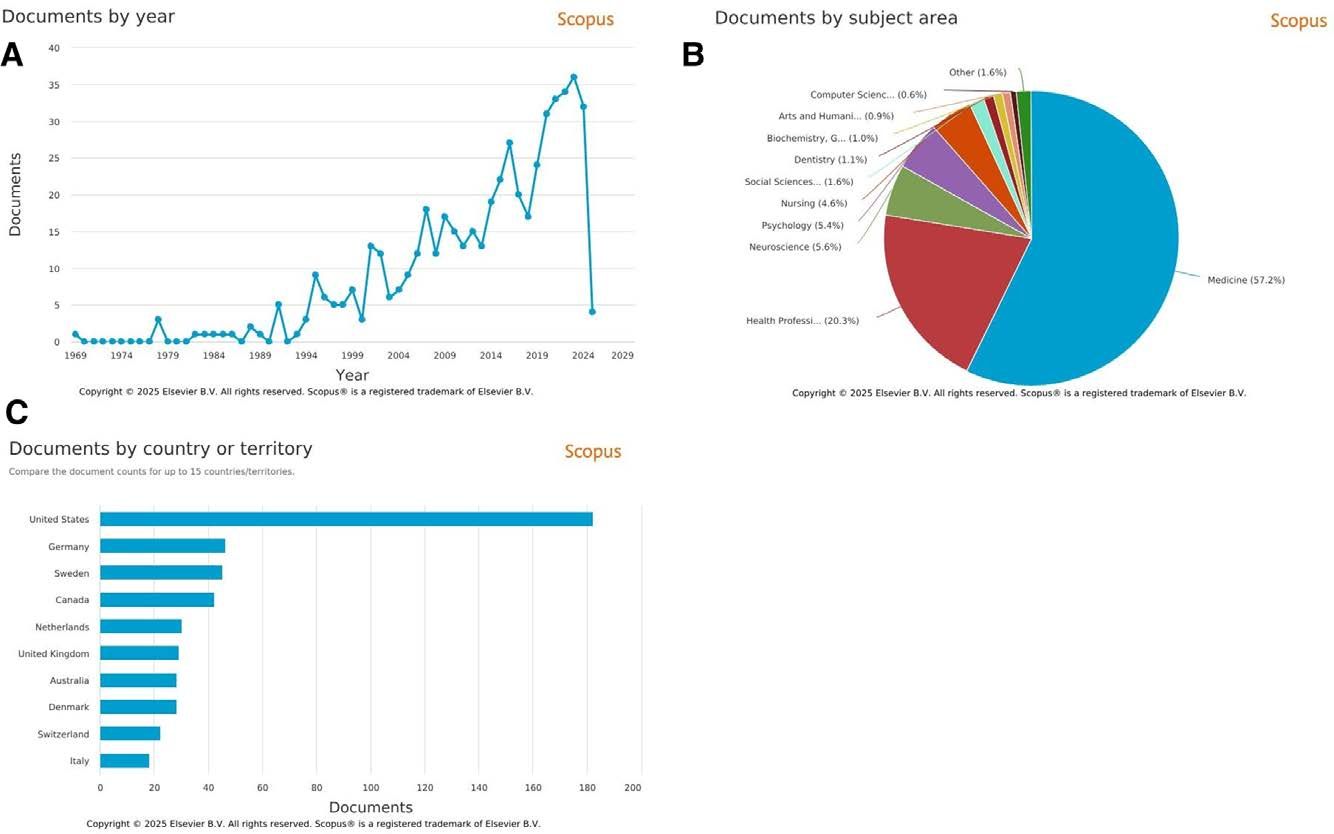
Analysis results of “Interdisciplinary Rehabilitation” on the Scopus database. (A) Number of publications per year. (B) Percentage of subject areas. (C) Number of publications per country.

Interdisciplinary rehabilitation is a theme that dates back to 1958 to coordinate the efforts of various professionals, with the ultimate goal of increasing rehabilitants’ satisfaction and improving their overall quality of life.^[2]^ In addition to evidence showing the benefits of collaborative practice for patient recovery and improvement, advancements in communication technology, increased awareness and recognition of other professionals, and co-location have made interdisciplinary work gaining popularity.^[3–7]^ This can be observed from the increasing publication trend in a brief search using the “interdisciplinary rehabilitation” keyword in the Scopus database (Fig. 1A). This trend has spanned multiple disciplines, mostly being medicine, followed by health professions, neuroscience, psychology, and nursing (Fig. 1B). As an international overview, this approach is becoming increasingly popular as it has been shown to be more effective than traditional, single-discipline approaches in rehabilitation, with the United States having the most publication output on the “interdisciplinary rehabilitation” theme followed by Germany and Sweden (Fig. 1C).

In terms of real-world implementation, interdisciplinary rehabilitation has been integrated into various national healthcare models. For instance, the United States has developed specialized interdisciplinary rehabilitation centers, such as the U.S. Department of Veterans Affairs Polytrauma Rehabilitation Centers, which provide coordinated care for brain injury and complex neurological conditions.^[8]^ In Sweden, interdisciplinary pain rehabilitation programs (IPRPs) are widely implemented and have shown significant effectiveness in improving functional outcomes.^[9]^ Similarly, in Germany, interdisciplinary stroke rehabilitation units are an integral part of post-acute care, ensuring smoother transitions from hospital to community-based recovery.^[10]^ Despite these advancements, interdisciplinary rehabilitation remains underutilized in many low- and middle-income countries, where access to a diverse team of rehabilitation professionals is limited. Future research should explore adaptable models that can be implemented in resource-constrained settings to maximize patient outcomes globally.

Nonetheless, many authors acknowledged the complexity of the conceptual distinction between “multidisciplinary” and “interdisciplinary” rehabilitation.^[11,12]^ According to Scott and Hofmeyer,^[11]^ while multidisciplinary rehabilitation “connects” the personnel involved in rehabilitation, interdisciplinary rehabilitation “coordinates” them. This coordination is particularly important with the ever-expanding specialties in healthcare and non-health related disciplines. The composition of the interdisciplinary rehabilitation team can vary across clinical settings. Singh and colleagues suggested that a typical team may comprise a rehabilitation physician, rehabilitation nurse, occupational therapist, physiotherapist, speech and language therapist, clinical psychologist, social worker, prosthetist, orthotist, rehabilitation engineer, and dietician.^[13]^

In this paper, we shed light on the extent of interdisciplinary rehabilitation among patients with neurological disorders, focusing on chronic pain, stroke, and traumatic brain injury on which the “interdisciplinary rehabilitation” theme has been mostly utilized, and highlight the implications for future research and clinical practice.

## 2. Interdisciplinary rehabilitation in chronic pain

Chronic pain is defined as pain that persists or recurs for more than 3 months.^[14]^ Due to the intractable nature of chronic pain conditions and the high risk of opioid dependence, there has been an increasing interest in exploring non-pharmacological interventions to better meet the needs of this vulnerable population. IPRPs are presently considered an evidence-based treatment for chronic pain.^[15,16]^ Research has demonstrated that interdisciplinary approaches can lead to better pain management outcomes, improved physical function, and enhanced quality of life for patients with chronic pain. These programs often incorporate a combination of medical treatments, physical therapy, psychological support, and lifestyle modifications to address the multifaceted nature of chronic pain.

According to a large recent study with real-world data, the analgesic efficacy of IPRPs was equally effective between non-neuropathic and neuropathic pain conditions, despite the high resistance to conventional pharmacological treatments of the latter.^[15]^ Furthermore, the IPRPs not only reduced the pain levels but also improved many of the pain-accompanying symptoms such as depression^[17–19]^ and insomnia.^[20,21]^

According to the International Association for the Study of Pain, an IPRP is a:

“Multimodal treatment provided by a multidisciplinary team collaborating in assessment and treatment using a shared biopsychosocial model and goals. For example: the prescription of an anti-depressant by a physician alongside exercise treatment from a physiotherapist, and cognitive behavioral treatment by a psychologist, all working closely together with regular team meetings (face to face or online), agreement on diagnosis, therapeutic aims, and plans for treatment and review.”^[22]^

A recent systematic review demonstrated that patient-centered IPRPs were more effective than usual care in alleviating chronic musculoskeletal pain.^[5]^ Other systematic reviews showed that IPRPs can improve functioning and quality of life in patients with various chronic pain conditions regardless of gender,^[16,23]^ but might vary, however, depending on the ethnoculturally diverse backgrounds.^[24]^

The need for additional professions in the IPRP is dynamic and varies depending on the population. For instance, the inclusion of language interpreters in the IPRP team was shown to be an efficient strategy in pain rehabilitation among immigrants with insufficient knowledge of the language.^[25]^ Whereas in pediatric populations, the parents were considered to have a crucial role in their children’s pain experience. Therefore, involving the parents in IPRP programs and improving their functioning and awareness of the child’s condition was linked to greater pain reduction after IPRP interventions.^[26]^ While in workers with chronic pain, the involvement of their employers in IPRP programs was shown to facilitate rehabilitation outcomes and return to work and reduce the sick leave of the workers.^[27–29]^ On the other hand, a novel discipline was proposed to be included in the IPRP, that is spiritual care and religious-oriented interventions.^[30]^ However, such spiritual discipline is still under-investigated in the rehabilitation field in general,^[31]^ and chronic pain in particular.^[30]^

Nonetheless, the wide-ranging disparity of reported outcomes in IPRP studies has presented difficulties for healthcare decision makers. Therefore, universal communication through unified outcome measures is needed to enhance the quality of evidence in future studies.^[32]^

## 3. Interdisciplinary rehabilitation in traumatic brain injury

Traumatic brain injury (TBI) represents a significant public health concern that could result in coma and death. Early interdisciplinary rehabilitation (EIR) is considered an effective approach in the neurointensive care unit for patients with moderate to severe TBI injuries. A recent systematic review found that EIR programs may improve functional outcomes and reduce socioeconomic costs among TBI patients.^[33]^ However, the criteria for selecting TBI patients in neurointensive care who would respond best to EIR remains debatable. Alvsåker et al^[34]^ found that the injury severity and the need for neurosurgery were the most important predictors for selecting patients in the EIR rehabilitation programs.

On the other hand, given the persistent post-concussion symptoms among mild TBI patients, interdisciplinary outpatient interventions also play an important role in the rehabilitation of these patients, for whom a 16-session interdisciplinary group outpatient rehabilitation intervention was shown to significantly improve performance and satisfaction ratings on the long-term.^[35]^

However, the optimal utilization of healthcare personnel in interdisciplinary practice is uncertain. For example, a recent scoping review indicated that although both occupational therapy and physiotherapy are crucial in rehabilitation, however, physiotherapy was frequently represented, whereas occupational therapy services were often overlooked in the management of traumatic brain injuries.^[36]^ This in turn raises the question of what defines and determines the best interdisciplinary practice in TBI.

## 4. Interdisciplinary rehabilitation in stroke

Stroke is a major cause of morbidity and mortality among elderly populations. Despite advances in early management, many stroke survivors suffer from long-term neurological and psychiatric sequelae.^[37]^ Findings from neurophysiological and histological studies have demonstrated an early critical time window during which the brain is more likely to be responsive to neurorehabilitation. This period is believed to span the first 3 months following stroke.^[38]^ Given the vascular origin of stroke incidents, an interdisciplinary collaboration between cardiovascular and neurological professionals is crucial to achieve better rehabilitation outcomes and reduce the occurrence of recurrent strokes.^[39]^ Apart from these 2 disciplines, the need for other professionals in interdisciplinary rehabilitation depends largely on the damaged brain region and subsequent neurological symptoms. For instance, a study showed that the presence of an interdisciplinary visual team in the acute and sub-acute stroke unit increased awareness of the existence of visual deficits among stroke patients and provided the necessary interdisciplinary assessment and rehabilitation.^[40]^ While another study recommended the use of an Interdisciplinary Stroke Assessment Battery in inpatient rehabilitation facilities to identify patients with acute stroke who are more likely to fall during their stay.^[41]^

The focus of interdisciplinary rehabilitation in stroke extends beyond the patient-physician interaction to involve the designing of the inpatient stroke rehabilitation facility itself. A framework for building such dedicated facilities was recently published with the contribution of interdisciplinary experts in both health and non-health related disciplines such as architectures, designers, and wayfinders.^[42]^ Beyond the inpatient rehabilitation process, it is not yet fully described how stable the effects of interdisciplinary rehabilitation are and how much clinical improvement can be seen in the time period following discharge. To address this knowledge gap, a multicenter study investigated the role of an Interdisciplinary Platform for Rehabilitation Research and Innovative Care of Stroke Patients (IMPROVE) in functional recovery beyond the inpatient rehabilitation treatment.^[43]^ This study found a significant improvement in the upper limb motor function and self-reported health status up to 1 year after the inpatient stroke rehabilitation.^[10]^ In addition, the same group found that the improvement in upper limb motor function and self-reported health status was associated with a higher pre-stroke socioeconomic status, presumably due to the higher utilization of outpatient therapies.^[44]^

## 5. Conclusions and future directions

In this paper, we aimed to highlight the trajectory of interdisciplinary rehabilitation studies in neurological conditions. These studies mainly focused on chronic pain, traumatic brain injury, and stroke. And while interdisciplinary rehabilitation programs are still in an early phase of development in traumatic brain injuries, they have reached an advanced stage in stroke rehabilitation and are already considered evidence-based in chronic pain management.^[16,45]^

Interdisciplinary rehabilitation has profound practical implications across various levels, impacting rehabilitants, professionals, healthcare systems, and society. On the patient’s level, interdisciplinary rehabilitation addresses the whole individual, rather than isolated symptoms. A team considers the individual’s physical, emotional, social, and vocational needs, leading to a more personalized and effective treatment plan. On the professional level, interdisciplinary rehabilitation fosters collaboration and communication among different disciplines, exposing professionals to different perspectives and approaches, which improves their professional development. In addition, many professionals find greater job satisfaction in working collaboratively and seeing the positive impact of interdisciplinary practices on their patients’ lives according to qualitative research studies.^[46]^ On the healthcare level, although the initial investment in interdisciplinary rehabilitation may be higher, it can lead to long-term cost-effectiveness by reducing hospital readmissions, decreasing the need for long-term care, and improving individuals’ ability to resume daily activities.^[47,48]^ While on the societal level, by promoting functional abilities and independence, interdisciplinary rehabilitation enables individuals with disabilities to participate more fully in society, contributing to a more inclusive and equitable community. In addition, it can reduce the burden on family caregivers by helping rehabilitants become more independent.^[45,49]^

From a policy perspective, there is a growing need to establish standardized interdisciplinary rehabilitation guidelines at the national and international levels. Countries with well-developed healthcare infrastructure, such as the United Kingdom and Australia, have incorporated interdisciplinary rehabilitation frameworks into their national stroke and chronic pain management guidelines. However, the lack of standardized protocols in other regions poses a barrier to widespread implementation. To bridge this gap, government health agencies and professional organizations should collaborate to develop comprehensive training programs for interdisciplinary teams, ensuring that rehabilitation professionals receive specialized education on collaborative care models. Additionally, hospital accreditation bodies could introduce interdisciplinary rehabilitation as a key performance indicator for patient-centered care, incentivizing hospitals to adopt these practices.

Nonetheless, numerous promising initiatives are currently under investigation to empower interdisciplinary rehabilitation programs. Among which, the Internet of Things is gradually entering the rehabilitation field to enhance connectivity between team members and represents a promising opportunity for developing smart rehabilitation systems in the e-Health area.^[50]^ Moreover, artificial intelligence chatbots are emerging as promising tools for exploring interdisciplinary subjects. A recent study found that ChatGPT-4 exhibited strong competency in addressing interdisciplinary rehabilitation inquiries by simulating multiple experts from complementary backgrounds.^[51]^ On the other hand, the role of the government and policymakers in consolidating interdisciplinary rehabilitation should never be underestimated. Examples of established government-supported initiatives in rehabilitation involve the NIH BRAIN initiative and the Helping to End Addiction Long-term^SM^ (HEAL) Initiative.^[52]^

Future research should focus on cost-effectiveness analyses to further support policymaking and investment in interdisciplinary rehabilitation. Moreover, public-private partnerships could facilitate the integration of emerging technologies, such as artificial intelligence-driven rehabilitation assistants and telehealth platforms, into interdisciplinary care, improving access in underserved areas. By addressing these gaps, interdisciplinary rehabilitation can evolve into a more universally accessible and effective healthcare model, improving patient outcomes worldwide.

## Author contributions

**Conceptualization:** Abdulhameed Tomeh, Muhammad Hibatullah Romli, Wan Aliaa Wan Sulaiman.

**Supervision:** Wan Aliaa Wan Sulaiman.

**Validation:** Abdulhameed Tomeh, Abdul Hanif Khan Yusof Khan, Zalina Abu Zaid, King-Hwa Ling, Liyana Najwa Inche Mat, Hamidon Basri, Ahmad Luqman Md Pauzi, Muhammad Hibatullah Romli, Wan Aliaa Wan Sulaiman.

**Visualization:** Abdulhameed Tomeh.

**Writing – original draft:** Abdulhameed Tomeh, Abdul Hanif Khan Yusof Khan, Zalina Abu Zaid, King-Hwa Ling, Liyana Najwa Inche Mat, Hamidon Basri, Ahmad Luqman Md Pauzi, Muhammad Hibatullah Romli, Wan Aliaa Wan Sulaiman.

**Writing – review & editing:** Abdulhameed Tomeh, Abdul Hanif Khan Yusof Khan, Zalina Abu Zaid, King-Hwa Ling, Liyana Najwa Inche Mat, Hamidon Basri, Ahmad Luqman Md Pauzi, Muhammad Hibatullah Romli, Wan Aliaa Wan Sulaiman.
